# Rac1b enhances cell survival through activation of the JNK2/c-JUN/Cyclin-D1 and AKT2/MCL1 pathways

**DOI:** 10.18632/oncotarget.7602

**Published:** 2016-02-22

**Authors:** Gang Li, Li Ying, Hong Wang, Si-Si Wei, Jie Chen, Yi-He Chen, Wei-Ping Xu, Qi-Qiang Jie, Qing Zhou, Yi-Gang Li, Yi-Dong Wei, Yue-Peng Wang

**Affiliations:** ^1^ Department of Cardiology, Affiliated Xinhua Hospital, Shanghai Jiaotong University (SJTU) School of Medicine, Shanghai, China; ^2^ Department of Neurology, Affiliated Xinhua Hospital, Shanghai Jiaotong University (SJTU) School of Medicine, Shanghai, China; ^3^ Department of Pediatrics, Affiliated Xinhua Hospital, Shanghai Jiaotong University (SJTU) School of Medicine, Shanghai, China; ^4^ Department of Cardiology, Shanghai Tenth People's Hospital, Tongji University School of Medicine, Shanghai, China

**Keywords:** Rac1b, cell proliferation, apoptosis, JNK2, AKT2

## Abstract

Rac1b is a constitutively activated, alternatively spliced form of the small GTPase Rac1. Previous studies showed that Rac1b promotes cell proliferation and inhibits apoptosis. In the present study, we used microarray analysis to detect genes differentially expressed in HEK293T cells and SW480 human colon cancer cells stably overexpressing Rac1b. We found that the pro-proliferation genes JNK2, c-JUN and cyclin-D1 as well as anti-apoptotic AKT2 and MCL1 were all upregulated in both lines. Rac1b promoted cell proliferation and inhibited apoptosis by activating the JNK2/c-JUN/cyclin-D1 and AKT2/MCL1 pathways, respectively. Very low Rac1b levels were detected in the colonic epithelium of wild-type Sprague-Dawley rats. Knockout of the rat Rac1 gene exon-3b or knockdown of endogenous Rac1b in HT29 human colon cancer cells downregulated only the AKT2/MCL1 pathway. Our study revealed that very low levels of endogenous Rac1b inhibit apoptosis, while Rac1b upregulation both promotes cell proliferation and inhibits apoptosis. It is likely the AKT2/MCL1 pathway is more sensitive to Rac1b regulation.

## INTRODUCTION

The small GTPase, Rac1, controls numerous cellular activities by cycling between an active GTP-bound form and an inactive GDP-bound form [1-3]. These processes are tightly regulated by three groups of proteins: guanine nucleotide exchange factors (GEFs), GTPase activating proteins (GAPs) and guanine nucleotide dissociation inhibitors (GDIs) [4, 5]. Activated Rac1 promotes cell cycle progression, angiogenesis, migration, transformation and cell survival [6-8]. Rac1 stimulates kinases such as p21-activated kinase (PAK), Jun NH2-terminal kinase (JNK) and p38 mitogen-activated protein kinase (MAPK) [9, 10], and the resulting signaling cascades lead to activation of AP1 transcription factor. Rac1 also stimulates other transcription factors, including NF-kB and serum response factor (SRF) [9-13]. Activation of these transcription factors leads to cyclin-D1 up-regulation, which promotes G1/S-phase progression.

Rac1 stimulates phosphatidylinositol-3 kinase (PI3K)-dependent activation of the AKT serine/threonine kinase, which suggests a role for Rac1 in anti-apoptotic signaling. Rac1 also stimulates the NADPH oxidase complex to generate reactive oxygen species, which up-regulates both cyclin-D1 and anti-apoptosis proteins [14-17]. Few mutations have been reported in Rac1, but Rac1 dysregulation has been associated with tumorigenesis [18-22].

Rac1b is an alternatively spliced form of Rac1, and is characterized by the insertion of an additional 19 amino acids (exon-3b) behind the intramolecular switch-II region [19, 23, 24]. This insertion induces conformational changes in switch-I and -II. Consequently, Rac1b exhibits impaired intrinsic GTPase activity, enhanced GEF-independent GDP/GTP exchange activity, and an inability to interact with Rho-GDI [25-27]. Thus, Rac1b exists in cells in a predominantly active GTP-bound form and triggers different downstream signaling cascades as compared to Rac1 [26-28]. Although it is reportedly transcribed in normal tissues, such as neurons, colon mucosa epithelial cells, thyroid and pancreatic ductal structures [23, 24], only low levels of Rac1b protein were detected in normal tissues adjacent to tumors of the thyroid [29] or lung [30], and in normal brain cortex in patients without cognitive impairment [31]. In contrast, Rac1b is upregulated in various malignant tumors including colorectal [24, 32-34], breast [19], lung [30, 35], thyroid [29] and pancreas [36, 37], and in Alzheimer's disease [31]. Rac1b upregulation is correlated with enhanced progression and poor prognosis [38, 39].

Rac1b promotes cell cycle progression and inhibits cell apoptosis, both of which are closely related to cell survival and tumorigenesis [33, 34, 40-42]. However, the underlying mechanisms of Rac1b in these processes are unclear. Both Matos, *et al.* [25] and Singh, *et al.* [27] reported that JNK was not involved in Rac1b-mediated cell proliferation. Matos, *et al.* showed that transient overexpression of Rac1b promoted phosphorylation and degradation of IkBα (an NF-kB suppressor), which stimulated NF-kB-mediated G1/S-phase progression and inhibition of apoptosis [25, 40]. Cichon, *et al.* also showed that Rac1b was upstream of NF-kB [39]. However, Singh, *et al.* found that Rac1b was unable to promote transcriptional activation of NF-kB or the subsequent up-regulation of cyclin-D1 [27]. In the Rac1b-mediated pro-proliferative pathway, the upstream cyclin-D1 regulators remain unclear. Aside from the NF-kB-mediated pathway [39, 40], Singh, *et al.* reported that Rac1b, similarly to Rac1, could activate AKT and NADPH oxidase [27, 43]. However, the downstream effectors involved in the Rac1b-related anti-apoptotic effect are still unknown.

In this study, we established HEK293T and human colon cancer SW480 cell lines stably overexpressing Rac1b and analyzed differentially expressed genes (DEGs) *via* microarray analysis. In both stable lines, overexpressing Rac1b activated/upregulated the JNK2/C-JUN/cyclin-D1 pathway to promote cell proliferation and the AKT2/MCL1 pathway to inhibit apoptosis. Very low Rac1b levels were detected in the colon epithelia of wild-type Sprague-Dawley (SD) rats. Knockout of the rat Rac1 gene exon-3b or knockdown of endogenous Rac1b in human colon cancer HT29 cells downregulated only the AKT2/MCL1 pathway. Our study reveals that very low levels of endogenous Rac1b inhibit apoptosis and upregulated Rac1b both promotes cell proliferation and inhibits apoptosis.

## RESULTS

### Establishment of stable cell lines overexpressing Rac1 or Rac1b

**Figure 1:**
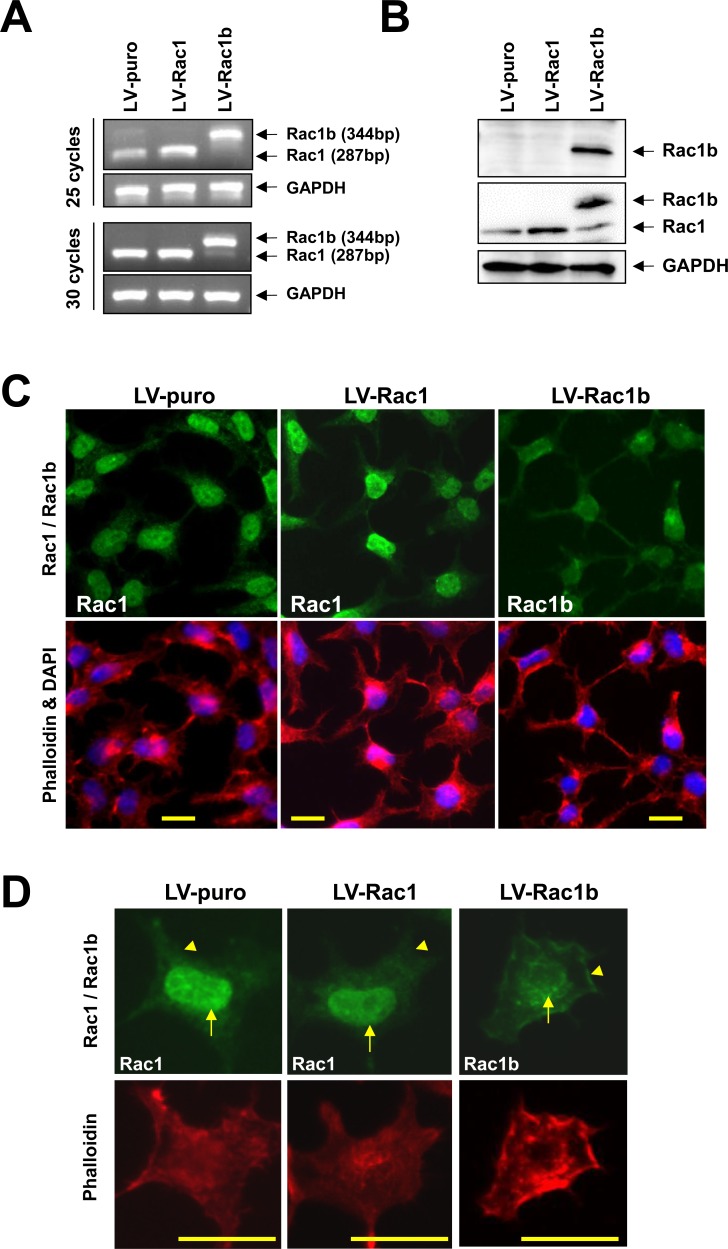
Stable HEK293T cell lines overexpressing Rac1 or Rac1b **A.** Total RNA from the cell lines was reverse transcribed into cDNA, followed by PCR amplification of endogenous and/or exogenous Rac1 and Rac1b transcripts. **B.** Whole cell lysate was analyzed by western blotting using anti-Rac1b (upper), anti-Rac1 (middle), and GAPDH (lower panel) antibodies. **C.** Immunofluorescence signals of Rac1/Rac1b (upper) and phalloidin/DAPI (lower panel) confirmed 100% infection efficiency. **D.** Distribution of Rac1 and Rac1b in the nucleus (arrows) and peripheral membrane (arrow head) among the three cell lines. Bars represent 20 mm.

Over-expression of Rac1 or Rac1b was confirmed by semi-quantitative RT-PCR. At 25 PCR cycles, LV-Rac1 cells demonstrated increased Rac1 transcript as compared with LV-puro cells. Rac1b transcript was detected in neither LV-puro nor LV-Rac1 cells, but was evident in LV-Rac1b cells (Figure 1A). Up to 30 cycles, Rac1 transcript differences narrowed between LV-puro and LV-Rac1 cells due to saturation, and a faint endogenous Rac1 transcript band was observed in Rac1b cells.

Rac1 protein was increased by 1.65(±0.16)-fold in LV-Rac1 cells as compared with LV-puro cells, similar to our previous report of a 2.0-fold change using a different lentiviral system [42]. Nascent Rac1b protein was only over-expressed in LV-Rac1b cells (Figure 1B). Endogenous and exogenous Rac1 localized mainly in the nucleus and cytoplasm, while exogenous Rac1b was observed mainly in the peripheral plasma membrane and cytoplasm (Figure 1C and 1D).

### Rac1b promotes cell viability and cell cycle progression during serum-starvation

**Figure 2:**
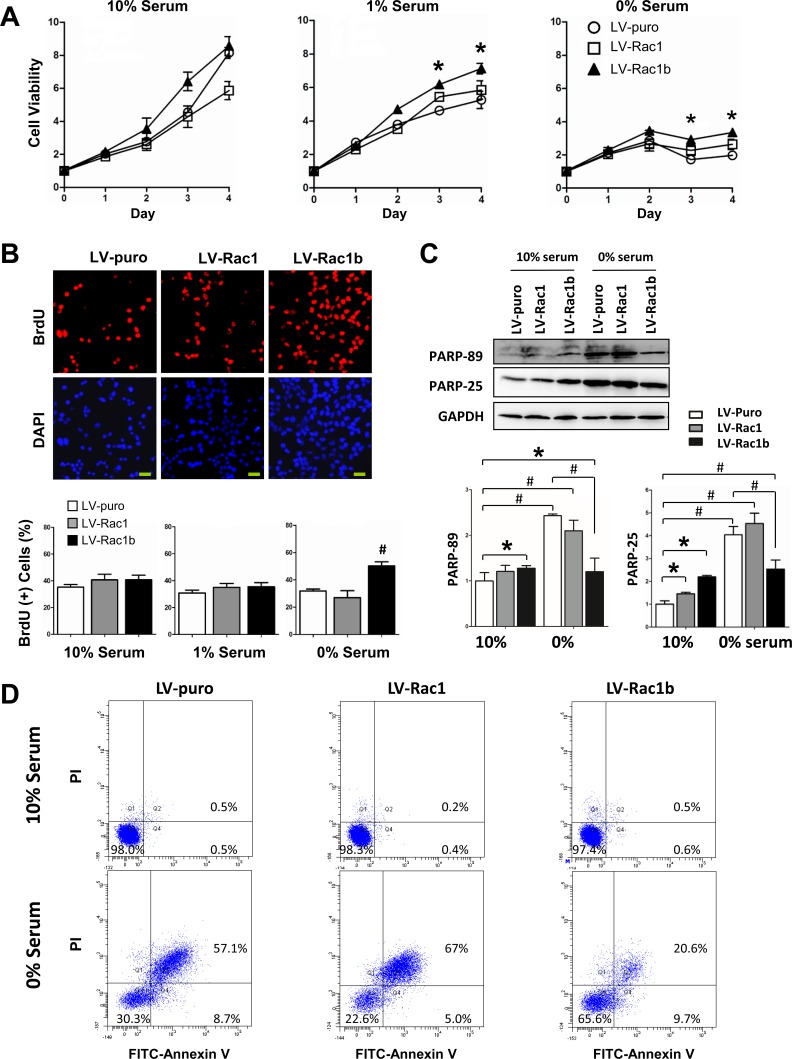
Rac1b promotes cell viability and cell cycle progression, and inhibits apoptosis **A.** Cells were cultured for 0-4 days in different serum conditions as indicated and cell viabilities were assessed daily using CCK-8 assay. Results are presented as OD values normalized to day 0. *p < 0.05 *vs*. LV-puro cells. **B.** Representative images of BrdU incorporation assay for three cell lines cultured in 0% serum. Of the total DAPI-stained blue cells (lower pannel), BrdU(+) cells were stained red (upper panel). Bars represent 20 mm. The percentages of BrdU(+) cells were compared in each serum condition. #*p* < 0.01 *vs*. LV-puro cells. **C.** Changes in cleaved PARP-89 and PARP-25 were compared in three cell-lines cultured in 10% and 0% serum. GAPDH was used as the control. Densities were first normalized to GAPDH and then to LV-puro cells in 10% serum. *N* = 5. *p < 0.05; #*p* < 0.01. Bars indicate comparisons. **D.** Representative results of flow cytometry in three cell lines cultured in 10% (upper) and 0% (lower panel) serum. Percentages for each cell line are labeled within each quadrant: Q-2 (late apoptosis), Q-3 (live), and Q-4 (early apoptosis).

To study the effects of Rac1 and Rac1b on cell survival, we cultured LV-puro, LV-Rac1, and LV-Rac1b cell lines in medium containing three different serum concentrations (10%, 1%, and 0%) for 4 days, and measured CCK-8 daily as an index of cell viability. There was no significant difference in viability among cells cultured in 10% serum (Figure 2A). In 1% serum, viability was slightly lower in all three lines. Rac1 cells showed a slightly higher but comparable viability compared to LV-puro cells. LV-Rac1b cells had the highest viability (*p* < 0.05). In 0% serum, cell viabilities were further reduced. Rac1b cells demonstrated a higher viability as compared with LV-puro cells (*p* < 0.05). There were no significant differences between LV-Rac1 and LV-puro cells, or between LV-Rac1b and LV-Rac1 cells (*p* > 0.05). These results indicated that Rac1b could enhance cell viabilities in both 1% and 0% serum conditions.

In medium containing 10% serum, all three cell lines showed similar BrdU incorporation levels. The percentages of BrdU(+) cells were 34.95±1.83% (LV-puro), 40.49±4.38% (LV-Rac1) and 40.99±3.26% (LV-Rac1b) (*p* > 0.05 between any two; Figure 2B). In 1% serum, all three cell lines showed slightly reduced but comparable BrdU incorporation levels (30.75±2.17% for LV-puro, 35.08±2.86% for LV-Rac1 and 35.55±3.07% for LV-Rac1b cells; *p* > 0.05 between any two). In 0% serum, as compared with LV-puro (32.66±1.38%) and Rac1 (27.21±5.04%) cells, Rac1b cells demonstrated a significantly increased percentage of BrdU(+) cells (53.74±4.42%, *p* < 0.01 for both), indicating that Rac1b could stimulate cell cycle progression under serum-starved conditions.

### Rac1b inhibits apoptosis during serum-starvation

We cultured cells in medium containing 10% serum for 48 h and analyzed cleaved PARP-89 and PARP-25 levels, two well-known apoptosis markers. Compared with LV-puro cells, both cleaved PARP-89 and PARP-25 were slightly increased in LV-Rac1 cells and moderately increased in LV-Rac1b cells (Figure 2C). However, Annexin-V assays demonstrated no significant differences in the percentages of apoptotic cells (sum of quadrant-2 and -4 on flow cytometry assay) among the three cell-lines (Figure 2D). Thus, Rac1b did not inhibit apoptosis at 10% serum.

We then cultured cell lines in medium with 0% serum for 48 h. Both PARP-89 and PARP-25 were similarly increased in LV-puro and LV-Rac1 cells, but not in LV-Rac1b cells (Figure 2C). Compared with LV-puro and Rac1 cells, Rac1b cells demonstrated a significant decrease in both PARP-89 and PARP-25 (*p* < 0.01). As shown in Annexin assays (Figure 2D), LV-Rac1b cells exhibited remarkably decreased apoptosis (31.5±3.7%; *p* < 0.01) as compared with LV-puro (58.3±6.2%) and LV-Rac1 (61.0±4.8%) cells. Taken together, these results demonstrated that Rac1b effectively inhibited serum starvation-induced apoptosis.

Evidence suggests that autophagy contributes greatly to cell survival and tumorigenesis. To determine if Rac1b sustains cell survival *via* upregulation of autophagy, we analyzed LC-3-II, an indicator of autophagosome formation. Autophagy took place at a low level in 10% serum and was upregulated in 0% serum. However, there were no significant differences among the three cell lines, indicating that Rac1b-mediated cell survival was not achieved through regulation of autophagy (data not shown).

### Rac1b-specific DEGs

Gene expression microarray analysis identified only 10 differentially expressed genes (DEGs) between LV-Rac1 and LV-puro cells (data not shown). In contrast, there were 482 DEGs between LV-Rac1b and LV-puro cells and 505 DEGs between LV-Rac1b and LV-Rac1 cells (Table S1). There was a high degree of overlap between the 482 and 505 DEGs (data not shown).

**Figure 3:**
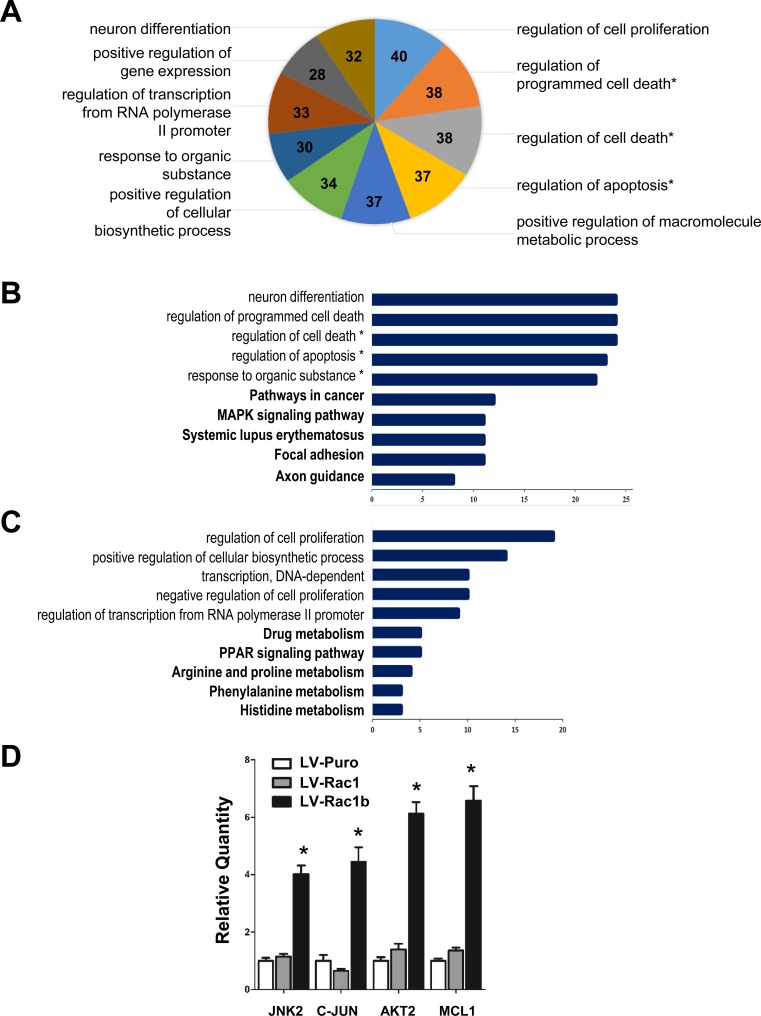
Rac1b-specific DEGs In total, 505 DEGs between LV-Rac1b and LV-Rac1 cells were identified by microarray screening and were clustered based on GO analysis. **A.** A total of 347 DEGs clustered within the top 10 Rac1b-mediated biological processes or signaling pathways. The number of clustered DEGs is labeled in each section of the graph. **B.** and **C.** The top 5 biological processes and signaling pathways (bold) in which up- and down-regulated genes clustered. *related to apoptosis. **D.** Validation of JNK, c-JUN, AKT2 and MCL1 by qPCR. Data are expressed as fold change compared to controls (LV-puro cells). *p < 0.05 *vs*. LV-puro and LV-Rac1 cells.

GO analysis of biological processes (mainly gene function) and KEGG analysis of biological processes and signaling pathways were applied to functionally classify the 505 DEGs using the Database for Annotation, Visualization and Integrated Discovery (DAVID) [50, 51]. Of the 10 most upregulated GO Biological Process Classification cellular functions (Figure 3A), one was linked to “regulation of cell proliferation” and three were linked to “regulation of programmed cell death,” “regulation of cell death” and “regulation of apoptosis.” Of the five most significantly upregulated biological processes, regulation of cell death or apoptosis accounted for three (Figure 3B). “Pathways in cancer” and “MAPK signaling pathway” were ranked the top two most significantly upregulated pathways, while “regulation of cell proliferation” was the most significantly downregulated biological process (Figure 3C). Rac1b overexpression appeared to mainly alter cellular processes or signaling pathways related to cell proliferation and apoptosis.

We found that JNK2 (2.34-fold) and c-JUN (2.16-fold), both part of the MAPK pathway that works to promote cell cycle progression, were upregulated, but JNK1 and JNK3 were not included in the 505 DEGs (Table S1). AKT-2 (2.16-fold) and MCL1 (2.12-fold), both part of the PI3K-AKT-MCL1 pathway that regulates apoptosis inhibition, were also upregulated, but AKT-1 was not on the list of 505 DEGs. Validation qPCR results showed that JNK2 (4.0±0.31), c-JUN (4.45±0.52), AKT2 (6.13±0.37) and MCL1 (6.58±0.48 fold) were indeed upregulated in LV-Rac1b cells but not in LV-puro and LV-Rac1 cells (Figure 3D). We hypothesized that these four upregulated proteins lead to activation of two cooperative pathways and underlie the mechanisms for Rac1b-mediated cell survival.

### Rac1b promotes cell cycle progression through the JNK2/c-JUN/cyclin-D1 pathway

**Figure 4:**
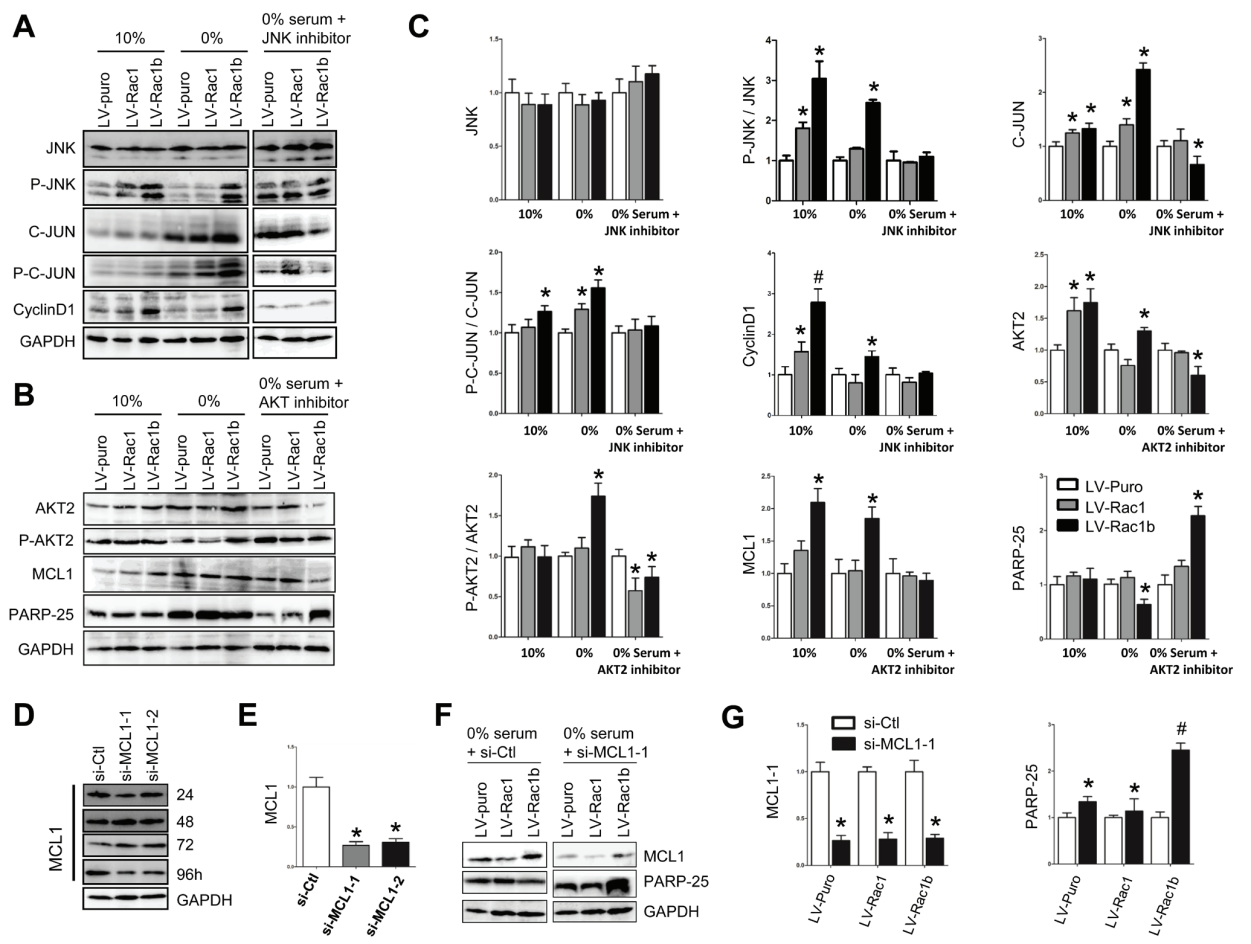
Rac1b promotes cell proliferation by up-regulation/activation of JNK/c-JUN/cyclin-D1 and inhibits cell apoptosis by up-regulation/activation of AKT2/MCL1 **A.** and **B.** Changes in JNK, p-JNK, c-JUN, p-c-JUN, cyclin-D1, AKT2, p-AKT, MCL1 and PARP-25 were compared by western blotting in the three cell-lines cultured in 10% and 0% serum, and 0% serum supplemented with JNK inhibitor (10 mM, 48 h; A) or AKT2 inhibitor (10 mM, 48 h; B). GAPDH was used as the control. **C.** Densities in A and B were first normalized to GAPDH and then to LV-puro cells in each serum condition. *N* = 3~5. *p < 0.05 and #*p* < 0.01 *vs*. LV-puro cells in each corresponding serum condition. **D.** Interfering effects of si-Ctl, si-MCL1-1 and si-MCL1-2 on MCL1 expression were compared 24-96 h after transfections in LV-puro cells. **E.** Summary of the interfering effects on MCL-1 at 96 h. *N* = 5. *p < 0.05 *vs*. si-Ctl. **F.** Changes in MCL1 and PARP-25 were compared by western blotting in the three cell-lines after transfection of si-Ctl (left) and si-MCL1-1 (right) for 96 h. **G.** Densities of MCL1-1 and PARP-25 in F were first normalized to GAPDH and then to the cell-lines transfected with si-Ctl. *N* = 4. *p < 0.05 and #*p* < 0.01 *vs*. si-Ctl.

As a known downstream effecter of Rac1, JNK could phosphorylate c-JUN, which interacts with c-fos to form transcription factor AP1 and stimulates cyclin-D1 expression. Inconsistent with the qPCR results (Figure 3D), JNK2 levels were not increased in LV-Rac1b cells cultured in either the 10% or 0% serum conditions (Figure 4A and 4C). In both 10% and 0% serum, p-JNK was slightly increased in LV-Rac1 cells and significantly increased in LV-Rac1b cells compared to LV-puro cells. JNK1/2 inhibitor SP-600125 (10 mM) completely abolished both Rac1 and Rac1b-induced JNK phosphorylation.

Downstream of JNK2, we measured both expression and phosphorylation of c-JUN (p-c-JUN). In 10% serum, activated JNK2 significantly upregulated c-JUN expression in both LV-Rac1 and LV-Rac1b cells, and p-c-JUN only in LV-Rac1b cells (Figure 4A and 4C). In 0% serum, activated JNK2 upregulated c-JUN expression and phosphorylation in both cell lines. SP-600125 inhibited JNK2-induced c-JUN upregulation and phosphorylation in LV-Rac1b cells.

Downstream of c-JUN, we measured expression of cyclin-D1, a key mediator of G1/S-phase progression (Figure 4A and 4C). In both 10% and 0% serum, cyclin-D1 expression was increased in LV-Rac1b cells, but not in LV-puro or LV-Rac1 cells. SP-600125 suppressed cyclin-D1 expression in LV-Rac1b cells, but not in LV-puro or LV-Rac1 cells. These results show that Rac1b upregulates JNK2/C-JUN/cyclin-D1 to promote G1/S-phase progression.

### Rac1b inhibits apoptosis by upregulating the AKT2-MCL1 pathway

Previous reports showed that MCL1 is downstream of AKT and could enhance cell survival by inhibiting apoptosis [47], but whether MCL1 is involved in Rac1b-mediated anti-apoptosis is unclear. We hypothesized that Rac1b's apoptosis inhibition was AKT2-MCL1-dependent.

In 10% serum, AKT2 was increased 1.62(±0.20)-fold in LV-Rac1 cells and 1.75(±0.22)-fold in LV-Rac1b cells compared with LV-puro cells (Figure 4B and 4C). In 0% serum, AKT2 was decreased 0.76(±0.09)-fold in LV-Rac1 cells, but increased 1.30(±0.05)-fold in LV-Rac1b cells. In 0% serum, CCT128930 (10 mM), a specific AKT2 inhibitor, significantly reduced AKT2 levels in LV-Rac1b cells, but not in LV-Rac1 cells.

p-AKT2 levels were not different among the three cell-lines in 10% serum (Figure 4B and 4C). In 0% serum, p-AKT2 was slightly elevated in LV-Rac1 cells, significantly elevated in LV-Rac1b cells, and was suppressed by CCT128930 in both cell lines.

In 10% serum, MCL1 was slightly upregulated in LV-Rac1 cells and significantly upregulated in LV-Rac1b cells compared with LV-puro cells (Figure 4B and 4C). In 0% serum, MCL1 was unchanged in LV-Rac1 cells but increased by 1.85(±0.17)-fold in LV-Rac1b cells. The increase in LV-Rac1b was completely abolished by CCT128930. As reflected by PARP-25 levels, CCT128930 promoted apoptosis in LV-Rac1b cells, but not in LV-Rac1 cells.

We then attenuated MCL1 expression using si-RNAs. Compared with scramble siRNA (si-Ctl), si-MCL1-1 reduced MCL1 expression most efficiently 96 h after transfection (27%±4%), while si-MCL1-2 was less potent (35%±4%) (Figure 4D and 4E). We transfected the three cell lines with si-Ctl and si-MCL1-1 and cultured them in 0% serum for 96 h. Apoptosis was increased in LV-Rac1b cells as compared with both LV-puro and LV-Rac1 cells (Figure 4F and 4G). These results indicated that suppression of downstream MCL1 could reverse Rac1b-mediated inhibition of apoptosis.

### Rac1b upregulates the JNK2/C-JUN/cyclin-D1 and AKT2/MCL1 pathways in human colon cancer cell lines

**Figure 5:**
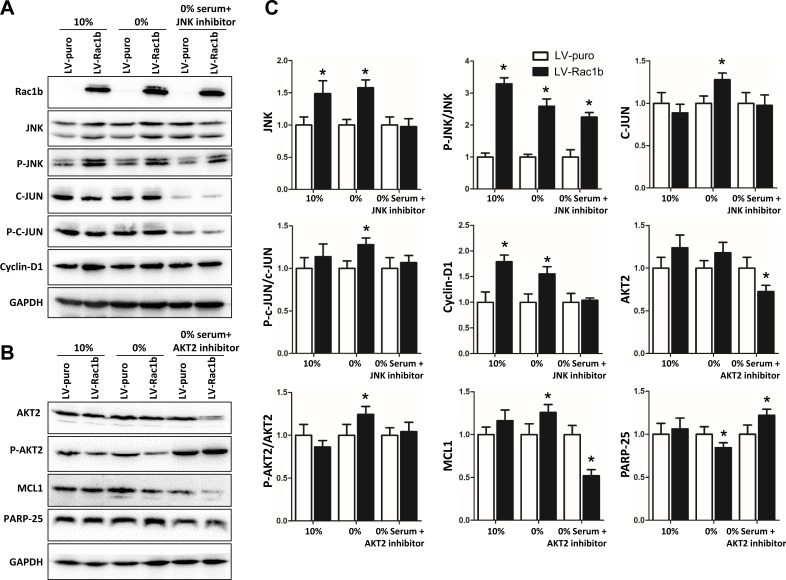
Rac1b upregulates and activates the JNK/c-JUN/cyclin-D1 and AKT2/MCL1 pathways in SW480 cells Two stable SW480 cell-lines, transfected with LV-puro or LV-Rac1b, were cultured in 10% and 0% serum, and 0% serum supplemented with a JNK (10 mM, 48 h; A) or ATK2 (10 mM, 48 h; B) inhibitor. **A.** and **B.** Changes in JNK, p-JNK, c-JUN, p-c-JUN, cyclin-D1, AKT2, p-AKT, MCL1 and PARP-25 were compared by western blotting. **C.** Densities in A and B were first normalized to GAPDH and then to LV-puro cells. *N*= 4-5. *p < 0.05 *vs*. LV-puro cells in each corresponding serum condition.

We chose the SW480 and HT29 cell lines, in which endogenous Rac1b is respectively absent and present [25, 32, 33, 54, 55], to study whether Rac1b-mediated pathway modulation also occurs in human colon cancer cells. We cultured SW480 cells infected with LV-puro or LV-Rac1b in 10% and 0% serum (Figure 5A and 5C). Both JNK and p-JNK were upregulated in LV-Rac1b cells in either 10% or 0% serum as compared to LV-puro cells, and this affect was blocked by SP-600125 in 0% serum. In contrast, c-JUN was unchanged in 10% serum and was slightly increased in 0% serum. Similarly, p-c-JUN was increased slightly in 0% serum, and this was inhibited by SP-600125. Cyclin-D1 increased in both 10% and 0% serum and this was inhibited by SP-600125 in 0% serum.

Although AKT2 levels were unchanged in LV-Rac1b cells in 10% and 0% serum, they were significantly reduced by the AKT2 inhibitor, CCT128930, when compared to LV-puro cells (Figure 5B and 5C). In contrast, p-AKT2 was unchanged in 10% serum, but mildly increased in 0% serum in LV-Rac1b cells. p-AKT2 was slightly increased by the AKT2 inhibitor in LV-Rac1b cells as compared with LV-puro cells. MCL1 was unchanged in LV-Rac1b cells in 10% serum, increased in 0% serum, and significantly decreased in LV-Rac1b cells after treatment of CCT128930. Similarly, PARP-25 demonstrated no significant change in 10% serum, a mild but significant decrease in 0% serum, and a significant decrease after treatment of CCT128930. These results indicated that overexpressed Rac1b promoted cell proliferation through the JNK2/C-JUN/cyclin-D1 pathway and inhibited cell apoptosis through the AKT2/MCL1 pathway in SW480 cells.

**Figure 6:**
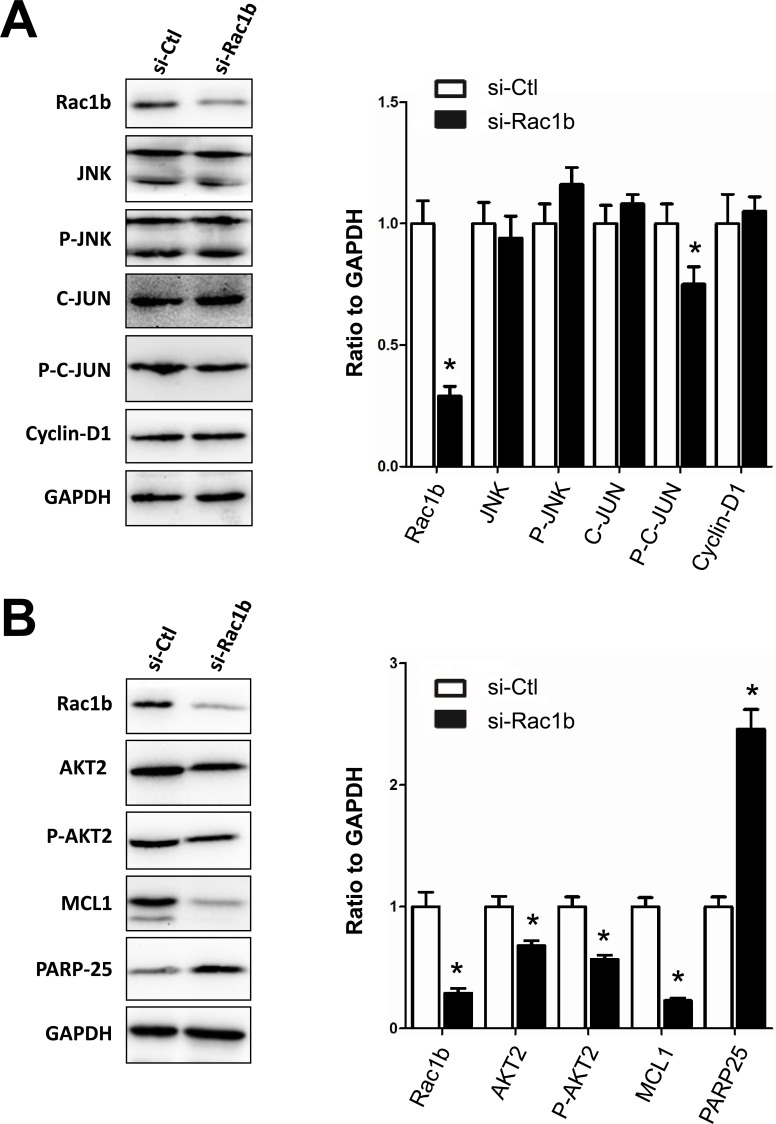
Rac1b upregulates and activates the AKT2-MCL1 pathway in HT29 cells HT29 cells were transfected with scramble siRNA (si-Ctl) or siRNA specific for endogenous Rac1b for 96 h. **A.** and **B.** Changes in Rac1b, JNK, p-JNK, c-JUN, p-c-JUN, cyclin-D1, AKT2, p-AKT, MCL1 and PARP-25 were compared by western blotting. Densities were first normalized to GAPDH and then to si-Ctl. *N* = 4. *p < 0.05 *vs*. si-Ctl.

We analyzed the JNK2/C-JUN/cyclin-D1 and AKT2/MCL1 pathways in HT29 cells with and without siRNA-knockdown of endogenous Rac1b (Figure 6A). siRNA knocked down Rac1b expression to about 29%±4% of the control level. Rac1b downregulation did not result in significant changes in JNK2, p-JNK, c-JUN, and cyclin-D levels, although p-c-JUN showed a significant decrease. However, Rac1b downregulation remarkably decreased AKT2, p-AKT2 and MCL-1 levels, and significantly increased PARP-25 (Figure 6B). These results indicated that while knockdown of endogenous Rac1b in HT29 cells did not affect the pro-proliferative JNK2/c-JUN/cyclin-D1 pathway, it dramatically attenuated the anti-apoptotic AKT2/MCL1 pathway.

### Rac1b-mediation of the JNK2/C-JUN/cyclin-D1 and AKT2/MCL1 pathways in normal colon mucosa epithelia in rat

**Figure 7:**
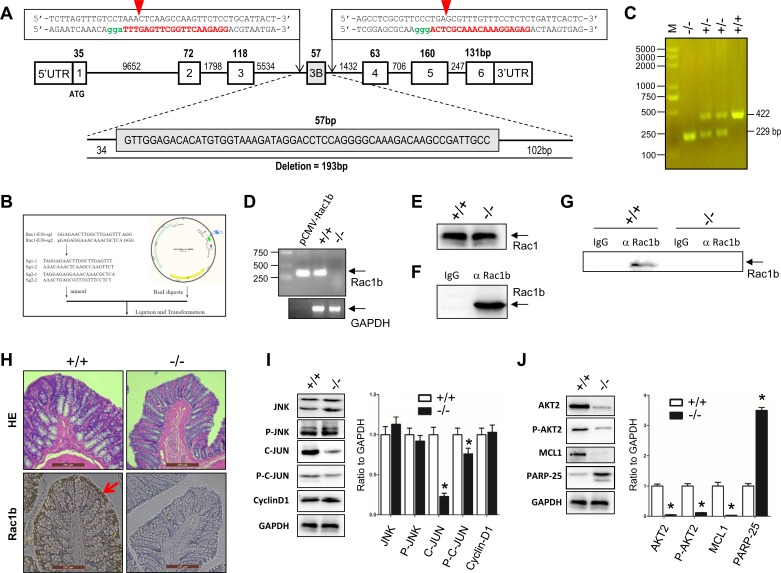
Generation of Rac1b^−/−^ SD rats and analysis of Rac1b-mediated AKT2/MCL1 pathway activation in the normal colon mucosa epithelia **A.** Diagram of the rat Rac1 gene with our double Cas-9 sequences used to delete 193 bp nucleotides covering the exon-3b. The red capital letters represent recognition sequences for sgRNAs and the green letters are PAM sequences. Red arrowheads illustrate the cutting sites for CRISPR/Cas9. **B.** The sgRNA sequences and structure of pUC57Kan-T7-gRNA for transcription of sgRNA. **C.** Three genotypes of the F2 rats. **D.** and **E.** RT-PCR of Rac1b-specific transcripts (D) and Rac1 protein (E) from the colon mucosa of Rac1b^+/+^ and Rac1b^−/−^ rats. **F.** Competence of Rac1b antibodies in immnoprecipitation. **G.** Immunoprecipitation of endogenous Rac1b in the colon mucosa of rats. **H.** Immunohistochemistry of HE and Rac1b in rat colon mucosa. **I.** and **J.** The colon mucosa, harvested from WT and Rac1b^−/−^ rats, was lysed in SDS-sample buffer. Changes in JNK, p-JNK, c-JUN, p-c-JUN, cyclin-D1, AKT2, p-AKT, MCL1 and PARP-25 were compared by western blotting. Densities were first normalized to GAPDH and then to +/+. *N* = 3. *p < 0.05 *vs*. Rac1b^+/+^.

Several studies reported that normal colon mucosa epithelia have Rac1b transcripts, but no Rac1b protein [25, 32, 54, 55]. To determine whether low levels of Rac1b expression could still promote the JNK2/C-JUN/cyclin-D1 and AKT2/MCL1 pathways, we knocked out a 193bp fragment including the entire exon-3b of the Rac1 gene in SD rats using the CRISPR/Cas9 technique (Figure 7A-7C). As detected by RT-PCR using colon mucosa, the Rac1b transcript was knocked out in Rac1b^−/−^ rats (Figure 7D). In contrast, both the Rac1 transcript (data not shown) and protein (Figure 7E) were not affected in the normal colon mucosa epithelia between age- and body weight-matched WT and Rac1b^−/−^ rats.

Rac1b protein was detected only at very low levels in the normal colon mucosa epithelia of WT rats by routine western blotting. We confirmed that our Rac1b antibodies were able to immunoprecipitate our purified recombinant 6his-Rac1b (Figure 7F). We detected low levels of endogenous Rac1b protein in the normal colon mucosas of WT SD rats by immunoprecipitation, but not in Rac1b^−/−^ rats (Figure 7G). Immunohistochemical analysis showed that Rac1b was found mainly in the colon mucosa epithelia in WT rats (Figure 7H).

**Figure 8:**
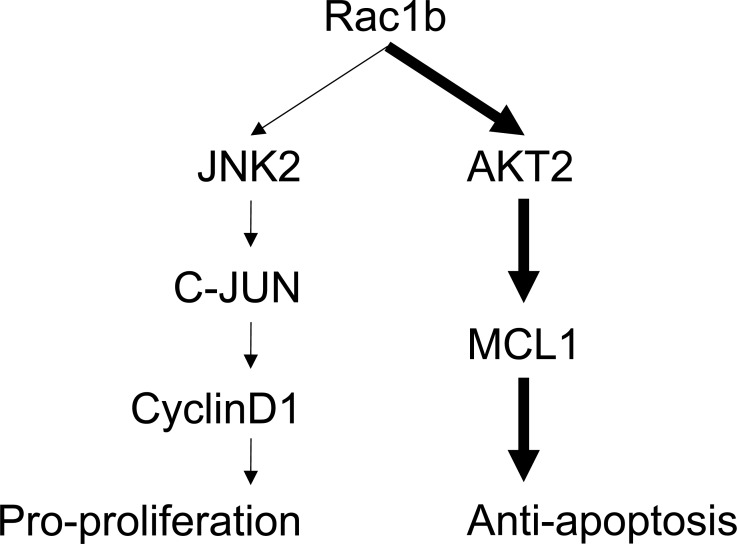
Schematic summary of Rac1b-mediated signaling in cell survival Bold arrows represent the major anti-apoptotic pathway.

We found no changes in JNK2, p-JNK or cyclin-D1 in the epithelia of colon mucosa between WT and Rac1b^−/−^ rats (Figure 7I). In contrast, c-JUN and p-c-JUN decreased significantly in Rac1b^−/−^ rats. These results indicated that the JNK2/c-JUN/cyclin-D1 pathway was not changed. However, AKT2, p-AKT2 and MCL-1 were significantly decreased, and PARP-25 was increased in Rac1b^−/−^ rats (Figure 7J). Thus, knockdown of low-level Rac1b mainly promoted apoptosis through the AKT2/MCL1 pathway. Our results indicated for the first time that low levels of Rac1b are important for inhibiting apoptosis in the normal colon epithelia. Figure 8 summarizes Rac1b-mediated pro-cell cycle progression and anti-apoptosis signaling.

## DISCUSSION

Rac1b is upregulated in many tumors [19, 29, 30, 33-37, 39], and we and others have reported that Rac1b enhances cell survival [33, 34, 40, 42]. However, the underlying mechanisms of this Rac1b function have not been fully elucidated. In this study, we made stable cell lines overexpressing Rac1b, confirmed Rac1b pro-proliferative and anti-apoptotic effects and identified Rac1b-specific DEGs. We found that: 1) Rac1b mainly regulates genes involved in cell proliferation and apoptosis; 2) Rac1b promotes cell cycle progression by activating JNK2, which subsequently stimulates c-JUN and up-regulates cyclin-D1; 3) Rac1b inhibits apoptosis by upregulating and activating the AKT2/MCL1 pathway; and 4) Rac1b expression level determines its impact on specific downstream pathways. At high levels, Rac1b stimulates both pro-proliferation and anti-apoptosis pathways. At low levels, it appears to only activate anti-apoptosis pathways.

Our microarray results demonstrate that there are only 10 DEGs between LV-Rac1 and LV-puro; much less than the 482 DEGs between LV-Rac1b and LV-puro and the 505 between LV-Rac1b and LV-Rac1. This may be explained by two mechanisms. First, as an important housekeeping gene, endogenous Rac1 is already redundant inside cells. Overexpression increased Rac1 levels by only about 1.6- to 2.0-fold above the basal level, both in this study (Figure 1B) and in our previous study using a different lentiviral system [42], and this is consistent with previous plasmid-mediated transient expression studies [26, 56]. Second, unlike constitutively-active Rac1b, Rac1 exists predominantly in a GDP-bound inactive form at resting state and its activation is tightly controlled. Thus, even with a 1.6- to 2.0-fold increase, Rac1 function may not be upregulated enough to result in observable and significant downstream effects as compared to controls.

Previous studies found that Rac1b activates cyclin-D1 transcription *via* NF-kB [32-34, 40] by inducing phosphorylation of the NF-kB inhibitor, IkB. However, Singh, *et al.* did not identify the role of NF-kB in the process [27]. Additionally, studies by Matos, *et al.* [25] and Singh, *et al.* [27] reported that Rac1b did not activate JNK and MAPK. Therefore, the upstream regulators of cyclin-D1 in this process were still unknown. Our microarray results revealed that Rac1b could significantly enhance transcription of JNK2 and c-JUN, both of which are part of the MAPK cascade and promote cell proliferation. However, Rac1b did not upregulate JNK2 protein. Further studies are needed to investigate whether Rac1b regulates JNK2 post-transcriptionally through an unknown feedback mechanism. Rac1b significantly enhanced JNK2 phosphorylation, which then activated downstream effector c-JUN. Rac1b not only activates c-JUN but also enhances c-JUN expression and subsequently upregulates cyclin-D1 expression. That JNK inhibition by SP-600125 attenuated cyclin-D1 expression in LV-Rac1b cells further suggests that Rac1b-mediated cyclin-D1 upregulation is mainly JNK2-dependent, but not c-JUN-dependent. Our results indicate that Rac1b-mediated JNK2-dependent cyclin-D1 upregulation governs the Rac1b pro-proliferative effect.

One possible explanation for the discrepancy between our result and those of previous reports is that both Matos and Singh over-expressed JNK1 [27, 57], but not JNK2. We found that only JNK2, but not JNK1 or JNK3, was a DEG in this study. Additionally, in the previous studies, Rac1b was overexpressed transiently by transfection of a plasmid, and overexpression was sustained for only a short period of time. The current study employed stable cell lines in which sustained Rac1b overexpression was comparable to Rab1b upregulation in tumor tissues, and was therefore better suited for evaluation of cellular functions.

Previous studies identified the role of activated AKT in Rac1b-mediated cell survival [27]. We found that AKT2, but not AKT1, plays a role in Rac1b-mediated inhibition of apoptosis. p-AKT2 levels in LV-Rac1b cells were higher than in LV-Rac1 cells. We speculated that Rac1 might work through AKT1 and Rac1b through AKT2. Further studies using specific phosphor-antibodies to distinguish p-AKT1 and p-AKT2 are needed to address this question. Downstream of AKT, four proteins, MDM2, Bad, Bak and Bax, are pro-apoptotic. Another three proteins, Bcl-xL, Bcl2 and MCL1 belong to the Bcl-2 family and are anti-apoptotic [44-49]. Of these seven molecules, only MCL1 was significantly upregulated in our study. MCL1, an anti-apoptotic protein highly expressed in various cancer cells, has been associated with progression in a number of malignant tumors [47]. Using an AKT2-specific inhibitor and siRNA-mediated MCL1 knockdown, we found that AKT2/MCL1 pathway activation was directly involved in Rac1b-mediated anti-apoptosis.

We also observed that Rac1b-mediated pathway activation depends on Rac1b levels. Rac1b expressed at high, lentiviral-induced levels promotes proliferation and inhibits apoptosis. These results help to explain the functions of Rac1b upregulation in various tumors [19, 29, 30, 32-39, 43], along with why Rac1b levels increase proportionally with enhanced malignancy [38, 39]. At low levels, Rac1b only activates the ATK2/MCL1 pathway and inhibits apoptosis. It is likely that the AKT2/MCL1 pathway is more sensitive to Rac1b regulation.

So far, several studies have detected Rac1b transcription in normal tissues, including colon mucosa epithelia, colon crypt epithelial cells, thyroid tissue, and pancreatic ductal structures [23, 24]. However, very low levels of Rac1b protein have only been detected in normal tissues adjacent to tumors [29-31]. Based on our immunoprecipitation results in rat cells, Rac1b expressed at even very low levels in normal colon mucosa epithelia is capable of moderating the AKT2/MCL1 pathway. We postulated that a low level of Rac1b exerts important function in normal cells, tissues or organs, due to Rac1b's constitutive activity and resistance to degradation [58]. However, the functional consequences of knocking out Rac1 gene exon-3b, especially changes in the incidences of tumors and inflammatory diseases, such as Crohn's disease, still need to be evaluated carefully in Rac1b^−/−^ rats.

## MATERIALS AND METHODS

### Antibodies and reagents

Mouse anti-Rac1 antibody was purchased from Transduction Laboratories (Lexington, KY, USA). Rabbit Rac1b antibody was generated as described previously [42]. Other rabbit antibodies were purchased from Cell Signaling (anti-LC3A/B-D3U4C, anti-JNK1/2, anti-phospho-JNK1/2, anti-c-JUN, anti-phosphor-c-JUN, anti-AKT2 and anti-MCL1; MA, USA), Biovision (anti-PARP89; Milpitas, CA, USA), Abcam (anti-PARP25, anti-cyclin-D1, anti-AKT1 and anti-phosphor-AKT2; Cambridge, MA, USA) and Proteintech (HRP-conjugated secondary antibodies; Chicago, IL, USA). SP-600125 (JNK1/2 inhibitor) and CCT128930 (AKT2 inhibitor) were purchased from Selleck Chemicals (USA).

### Lentiviral construct cloning and packaging

Human Rac1 and Rac1b cDNA were cloned into the pHBLV-CMVIE-Puro vector as described previously [42]. Lentivirus (Lenti-puro, Lenti-Rac1, Lenti-Rac1b) packaging and concentration were performed by co-transfecting constructs with two helper plasmids, PSPAX2 and PMD2G, in HEK293T cells with technical help from Hanheng Biotech Co., Ltd (Shanghai, China).

### Cell culture, viral infection and siRNA transfection

HEK293T cells were cultured as described previously [42]. SW480 and HT-29 cells were cultured and passaged in Dulbecco's modified Eagle's medium (DMEM; HyClone, GE Healthcare Life Sci, Logan, UT, USA) supplemented with 4500mg/L high glucose, 10% fetal bovine serum, 100 units/ml penicillin, and 100 mg/ml streptomycin at 37°C and 5% CO_2_. HEK293T and SW480 cells at 50-70% confluence were infected with lentivirus (Lenti-puro, Lenti-Rac1, Lenti-Rac1b) with 5 mg/ml polybrene (Sigma). Cells were selected using 2 mg/ml puromycin. For siRNA transfection, cells at 50-70% confluence were transfected with 50nM MCL1 or Rac1b siRNA (5′ → 3′; si-MCL1-1: AAAAUAUCAACUAAGAUCCUU; si-MCL1-2: AGGAAUUGAUAUAAAUAUCUG; si-Rac1b-1: CAGUUGGAGAAACGUACGGTT; si-Rac1b-2: CGUACGGUAAGGAUAUAACTT) or scramble siRNA (AAGCGCGCUUUGUAGGAUUTT; RiboBio, China) using lipofectamine-2000 (Invitrogen, USA). Interference efficiencies were checked daily for 24-96 h after transfection.

### RNA isolation and quantitative real-time PCR

Total RNA isolated from the three stable cell lines using TRIzol reagent (Invitrogen, USA) was transcribed into cDNA using the 5×PrimeScript RT Kit (Takara, Japan). cDNA was measured by semi-quantitative PCR or quantitative RT-PCR (qPCR) with THUNDERBIRD SYBR qPCR Mix (Toyobo, Japan). The following primer pairs (5′ → 3′) were used. Rac1/Rac1b F: TGCCAATGTTATGGTAGATGG and R: TGGGAGTCAGCTTCTTCTCC; JNK2

F: TACGTGGTGACACGGTACTACC and R: CACAACCTTTCACCAGCTCTCC; c-JUN F: CCTTGAAAGCTCAGAACTCGGAG and R: TGCTGCGTTAGCATGAGTTGGC; AKT2 F: CATCCTCATGGAAGAGATCCGC and R: GAGGAAGAACCTGTGCTCCATG; MCL1 F: CCAAGAAAGCTGCATCGAACCAT and R: CAGCACATTCCTGATGCCACCT; and GAPDH F: TCTTCACCACCATGGAGAAG and R: TGACCTTGCCCACAGCCTTG.

### Cell proliferation assay

Stable cell lines, seeded in 96-well plates at 3×10^3^ cells/well in medium containing 10% serum, were cultured overnight. Cells were then cultured in 10%, 1% or 0% serum conditions. Ten μl of Cell Counting Kit-8 (Dojindo, Japan) reagent was added to each well and plates were incubated at 37°C for 2 h. Optical density (OD) was measured at 450nm using a microplate reader (BioTek, USA). Cell proliferation rate was calculated as: [(OD experiment - OD blank) / (OD 1^st^ day - OD blank)] x 100%. All experiments were performed in triplicate and repeated three times [42].

### BrdU incorporation assay

Cells were cultured on 10×10mm polylysine-coated cover slips, incubated with 60 μM BrdU (Sigma) for 12-16 h, fixed with cold acetone-methanol (1/1, v/v), denatured with 4M HCl, and then neutralized with 1M Tris-HCl (pH 8.0). Next, cells were incubated with mouse anti-BrdU antibodies (Sigma), Cy3-labeled secondary antibody (Jackson Laboratory, USA) and 1 μg/ml DAPI (Dojindo; Japan), and were mounted with Antifade medium (Molecular Probes, OR). BrdU(+) cells were visualized by fluorescence imaging on a Leica DMI3000B microscope [42].

### Apoptosis assay

Cells cultured for 2 days in 60-mm plates in 10%, 1% or 0% serum conditions were collected and washed twice with PBS. Cells were suspended in 500 μl binding buffer, double stained with Annexin V-FITC and Propidium Iodide (KeyGEN, China) in the dark for 10 min, and analyzed by flow cytometry (BD, USA).

### Microarray

Total RNA preparation from the three stable HEK293T cell lines, quality control, reverse transcription, cDNA labeling, hybridization, image acquisition, and analysis were performed by Oebiotech (Shanghai, China). Briefly, the cDNA was coupled to fluorescence dye and hybridized in four replicates to the Prime View™ Human Gene Expression Array (Affymetrix, USA). Data was normalized using the quantile algorithm by Genesrping software (version 12.5; Agilent Technologies). DEGs were identified through fold changes (≥2.0 as upregulated and ≤-2.0 as down-regulated) as well as *p*-values (≤0.05), followed by GO and KEGG analysis [50, 51].

### Western blotting and immunofluorescence

For western blotting, cells were rinsed with cold PBS, fixed with 10% trichloroacetic acid (Sigma) in PBS containing 2mM EDTA and 10mM dithiothreitol, washed in cold acetone containing 2mM dithiothreitol, air-dried, solubolized in SDS-sample buffer, sonicated, and subjected to SDS-PAGE. Immunofluorescence was analyzed as described previously [52, 53].

### Rac1b gene knockout rat

Target sites upstream and downstream of Rac1 gene exon-3b were obtained from the rat genome using the online CRISPR design tool (http://crispr.mit.edu). Two oligomer pairs for the generation of sgRNA expression plasmids were annealed and cloned into the BsaI restriction site of pUC57-sgRNA (Addgene, 51132; Figure 7B). DNA was amplified for the detection of T7 promoter and sgRNA regions and the PCR products were purified before being transcribed using the MEGAshortscript Kit (Ambion, AM1354) and purified using the miRNeasy Micro Kit (Qiagen, 217084). Cas9 expression vector (Addgene, 44758) was linearized with PmeI and transcribed using the T7 Ultra Kit (Ambion, AM1345). mRNA was purified using the RNeasy Mini Kit (Qiagen, 74104) according to the manufacturer's instructions. One-cell embryos were co-injected with Cas9 and sgRNA mRNA using the FemtoJet 5247 microinjection system under standard conditions. Tail biopsies of the founder rat were collected to extract genomic DNA. Products from PCR amplification of the target region were subjected to T7 endonuclease 1 (T7EN; NEB, M0302) cleavage assay. Briefly, a 422-bp DNA fragment containing the sgRNA target site was PCR-amplified with the following primers: Rac1b-F (TATGCGACTGCAGCTTTGGA) and Rac1b-R (CTTCCGGACACCCTCCTTTC). The PCR product was purified, denatured and reannealed in NEB Buffer 2 (NEB). Hybridized PCR products were digested with T7EN for 45 min and analyzed by 2% agarose gel. The presence of mutant Rac1b alleles in tail samples was confirmed by sequencing PCR products using the primers, Rac1b-Seq-F (TGCAGCTTTGGATTCCTCTG) and Rac1b-Seq-R (AAGCAGCTCGACCACTTTAC).

### Immunohistology and Immunoprecipitation

The animal study was approved by the Ethics Committee of Xinhua Hospital. All animal experiments were carried out in accordance with the NIH Guide for the Care and Use of Laboratory Animals. The rats were deeply-anesthetized with pentobarbital sodium (50 mg/kg, i.p.) and whole colons were excised and divided into three segments: ascending, transverse and descending. Each segment was cut in half, weighed and rinsed in ice-cold PBS. One half of each segment was fixed in 10% formalin, embedded in paraffin, sectioned at 5 microns, and stained with hematoxylin and eosin (H&E) and Rac1b. The second half was pulverized using a tissue-tearor on ice, with 100x cocktail protease inhibitors. Smashed tissue was immediately suspended in ice-cold TLB buffer containing protease inhibitors and sonicated [52]. Supernatant was obtained by centrifugation at 14,000g for 20 min and protein concentrations were measured using the Bradford method. Supernatants underwent pre-absorptions by protein-G beads (Roche; Indianapolis, IN, USA) for 1 h at 4°C. After removal of the beads, supernatants (1000 mg of total protein) were mixed with Rac1b antibodies (3 mg) for 4 h with rocking at 4°C. Protein-G beads were then added with an additional 4 h of rocking. Immunoprecipitates were centrifuged at 800g for 5 min at 4°C, washed four times with TLB buffer, and then resuspended in 60 ml of SDS sample buffer.

### Statistics

Data are expressed as means ± S.E. ANOVA tests and Student's *t*-tests were used to determine statistical significance. *P* < 0.05 was regarded as statistically significant.

## SUPPLEMENTARY TABLE


